# Characteristics, Antioxidant Activity Stability, and Anti-Fatigue Activity of Hydrolysates from *Cucumaria frondosa* Tentacles

**DOI:** 10.3390/molecules30040889

**Published:** 2025-02-14

**Authors:** Mingbo Li, Juan Chen, Qiuting Wang, Chuyi Liu, Wenkui Song, Leilei Sun

**Affiliations:** 1 Yantai Key Laboratory of Characteristic Agricultural Bioresource Conservation & Germplasm Innovative Utilization, School of Life Sciences, Yantai University, Yantai 264005, China; limingbo1711@163.com (M.L.); wqt1981979471@163.com (Q.W.); 2 College of Health, Yantai Nanshan University, Yantai 265713, China; chenjuan8796@126.com; 3 Marine Biomedical Research Institute of Qingdao, Qingdao 266073, China; liucy@ouc.edu.cn; 4 College of Food Science and Engineering, Ocean University of China, Qingdao 266003, China; 5 Guangdong Provincial Key Laboratory of Aquatic Products Processing and Safety, National Research and Development Branch Center for Shellfish Processing (Zhanjiang), Guangdong Provincial Engineering Technology Research Center of Seafood, Guangdong Province Engineering Laboratory for Marine Biological Products, College of Food Science and Technology, Guangdong Ocean University, Zhanjiang 524088, China; songwk@gdou.edu.cn

**Keywords:** *Cucumaria frondosa* tentacle, hydrolysate, characteristic, antioxidant activity, anti-fatigue activity

## Abstract

This study aimed to assess the impact of alcalase, trypsin, flavourzyme, and neutrase on the characteristics, antioxidant activity stability, and anti-fatigue activity of hydrolysates derived from *Cucumaria frondosa* tentacles (CFTHs). The results demonstrate that favourzyme hydrolysates exhibited the highest degree of hydrolysis (DH). Zeta potential and particle size measurements indicated that hydrolyzed peptides treated with favourzyme appeared aggregated and exhibited larger particle sizes. The antioxidant properties of CFTHs demonstrated good thermal stability, pH stability, and enhanced simulated gastrointestinal digestive stability. The anti-fatigue activity of CFTHs was examined using an acute exercise fatigue model. The results indicate that CFTHs extended the exhaustive swimming time of mice to 17.81 min. Additionally, CFTHs significantly elevated (*p* < 0.01) blood glucose (Glu) and liver glycogen (LG) levels, while also decreasing (*p* < 0.05) the concentrations of metabolites such as lactic acid (LA), urea nitrogen (BUN), creatine kinase (CK), lactate dehydrogenase (LDH), and ammonia (NH3). This reduction contributed to the alleviation of fatigue in the body. Furthermore, the levels of superoxide dismutase (SOD) and glutathione peroxidase (GSH-Px) were significantly increased (*p* < 0.05), which aided in mitigating oxidative damage induced by strenuous exercise. These findings strongly support the potential utilization of CFTHs in food products as natural antioxidant and anti-fatigue alternatives.

## 1. Introduction

Currently, humanity is confronting the challenges posed by COVID-19 [1], while the fast-paced lifestyle has led to various health issues, including fatigue, low immunity, anxiety, insomnia, and various chronic diseases such as hypertension, diabetes, obesity, and even cancer. As a result, there is an increasing emphasis on enhancing and maintaining good health. Fatigue, which can escalate to exhaustion, is typically triggered by a significant decrease in energy during physical exertion [2]. High-intensity and excessive exercise generally result in the production and accumulation of substantial amounts of free radicals, which are closely associated with oxidative damage and fatigue [3]. The antioxidant activities of peptides have been extensively investigated, with numerous studies emphasizing the potential for generating antioxidant peptides through the in vitro enzymatic digestion of marine protein resources. Liu et al. [1] confirmed that abalone visceral peptides exhibited significant in vitro antioxidant activity and cytoprotective effects against oxidative damage. Research has demonstrated that food-derived bioactive peptides can also exert their anti-fatigue effects by increasing glycogen reserves, enhancing the activity of antioxidant enzymes, reducing the accumulation of metabolites, and protecting tissue cells. Ye et al. [4] prepared peptides from the sea cucumber (*Stichopus japonicus*) and suggested that these peptides exhibited anti-fatigue activity in an endurance swimming rat model. They proposed that the anti-fatigue effects may be attributed to the normalization of energy metabolism, as well as the alleviation of oxidative damage and inflammatory responses.

*Cucumaria frondosa*, commonly referred to as the orange-footed sea cucumber, is the most abundant and widely distributed species in the cold waters of the North Atlantic Ocean, and is classified within the Cucurbitaceae family. *Cucumaria frondosa* is of commercial significance in eastern Canada [5]. It can grow to a maximum length of 40 to 50 cm, a width of 10 to 15 cm, and a weight ranging from 100 to 500 g. This species is a suspension feeder that employs its tentacles to capture phytoplankton, zooplankton, and organic materials. Certain enterprises market the dehydrated tentacles, referred to as “flower” [5]. *Cucumaria frondosa* thrives in a distinct growth environment and possesses a prolonged growth cycle, resulting in minimal pollutant accumulation and a rich nutrient profile [6]. Consequently, its quality is superior to that of other sea cucumber varieties. Following capture, *Cucumaria frondosa* is typically processed into various forms, including ready-to-eat sea cucumbers, dried sea cucumbers, and salted sea cucumbers. The primary product obtained from sea cucumbers is the body wall. This processing generates substantial by-products such as tentacles, intestines, and ovum, constituting approximately 50% of the sea cucumber’s total weight. Currently, the by-products of *Cucumaria frondosa* are entirely discarded, leading to environmental pollution and a waste of resources [7]. Hossain et al. [8] conducted a study on the free, esterified, and insoluble-bound phenolics found in the tentacles of *Cucumaria frondosa* and their antioxidant activity. However, there is a paucity of research regarding the structure and biological activity of its protein hydrolysates. Consequently, future research should focus on the utilization of *Cucumaria frondosa* by-products for the extraction of value-added products and their potential applications as nutraceuticals and functional food ingredients.

The physicochemical properties and biological activity of hydrolysates are predominantly influenced by the specific proteases employed and the method of hydrolysis applied [9,10]. Nevertheless, there is limited research on the effects of various proteases on the physicochemical and biological activities of hydrolysates derived from *Cucumaria frondose* tentacles. Therefore, this research aimed to explore the influences of different proteases on the physicochemical properties and antioxidant characteristics of hydrolysates derived from *Cucumaria frondose* tentacles (CFTHs), as well as their stability in antioxidant activity. Additionally, the research assessed the anti-fatigue effects of CFTHs by investigating their impact on biochemical indicators, including exhaustive swimming time, energy reserves, related metabolite accumulation, and oxidative stress in mice. The findings offer theoretical support for the utilization of by-products derived from *Cucumaria frondose*.

## 2. Results and Discussion

### 2.1. Degree of Hydrolysis of Hydrolysates Derived from Cucumaria frondosa Tentacles

**Figure 1:**
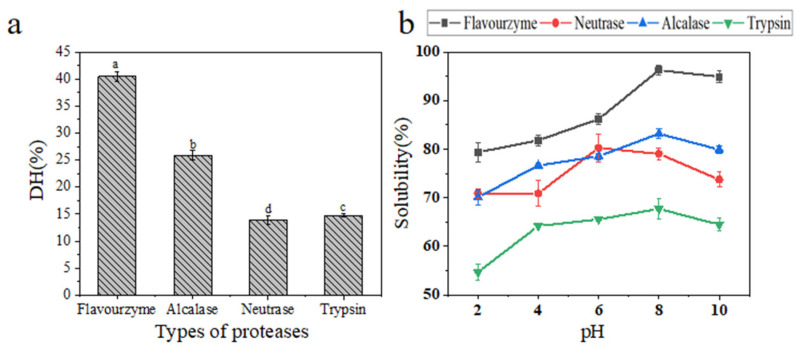
(**a**) DH of the CFTHs obtained by different proteases; (**b**) solubility of the CFTHs obtained by different proteases (n = 5). Different letters indicate significant differences (*p* < 0.05).

The hydrolysis of CFTHs was investigated using various proteases, and the degree of hydrolysis (DH) of CFTHs is illustrated in Figure 1a. The type of protease employed exhibited a significant impact on the DH of the product (*p* < 0.05) [11]. As shown in Figure 1a, the DH achieved with flavourzyme was significantly higher compared to the other three proteases. The results indicate that, with the exception of flavourzyme, the DH attained by the other proteases was below 30%. The difference arises from the fact that flavourzyme functions as both exopeptidases and endopeptidases, specifically as a cysteine protease with leucine aminopeptidase activity [12].

### 2.2. Solubility of Hydrolysates Derived from Cucumaria frondosa Tentacles

The solubility of protein hydrolysates is a critical factor that significantly influences their physicochemical and functional characteristics [13]. As illustrated in Figure 1b, among all the samples, the flavourzyme hydrolysate exhibited the highest solubility, while the solubility of the other three protease hydrolysates also surpassed 50%. The lower solubility observed in the trypsin hydrolysate may be attributed to its lower DH. This finding aligns with those of the study reported by Hau et al. [14], which suggested that a higher DH corresponded to smaller hydrolysate sizes. Smaller peptide molecules tend to form stronger hydrogen bonds with water compared to intact proteins, resulting in the increased solubility of CFTHs. Interestingly, Senadheera et al. [15] indicated that protein hydrolysates derived from different parts of the North Atlantic sea cucumber exhibited similar solubility characteristics. Notably, solubility improves with a shift towards more basic pH conditions.

### 2.3. Particle Size, Polydispersity Index, and Zeta Potential

**Figure 2:**
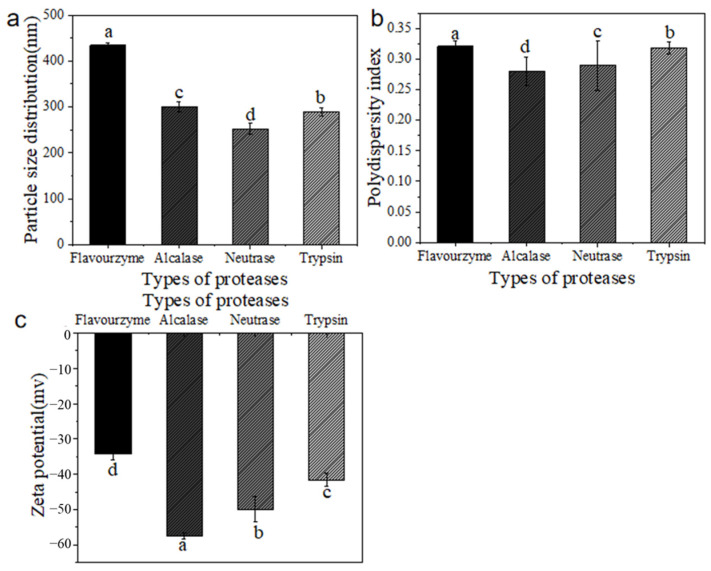
(**a**) The particle sizes of CFTHs obtained by different proteases; (**b**) the PDI of CFTHs obtained by different proteases; (**c**) the zeta potential of CFTHs obtained by different proteases (n = 5). Different letters indicate significant differences (*p* < 0.05).

The particle size, polydispersity index (PDI), and zeta potential of each hydrolysate are exhibited in Figure 2. Among the proteases examined, flavourzyme exhibited the largest particle size, whereas neutrase demonstrated the smallest. Contrary to expectations that the hydrolysate from flavourzyme would yield a smaller particle size compared to the other proteases, Figure 2 indicated that it was actually the largest. This phenomenon may be attributed to the aggregation of peptides and amino acids into larger fragments or particles due to the formation of chemical bonds [16].

The PDI serves as a crucial metric for assessing the dispersion properties of macromolecular polymers. A lower PDI value indicates improved dispersion in water [17]. Various protease treatments resulted in distinct dispersion characteristics among the hydrolysates, with flavourzyme demonstrating the most effective dispersion.

Furthermore, a higher absolute value of zeta potential diminishes the flocculation and aggregation caused by attractive forces, thereby enhancing the stability of the solution [18]. The hydrolysates derived from alcalase exhibited significantly greater absolute zeta potentials compared to the other treatments (*p* < 0.05). An elevated zeta potential promotes electrostatic repulsion between molecules and facilitates the diffusion of the hydrolysate, ultimately leading to improved dispersion [19].

### 2.4. Effects of Protease on Antioxidant Activity of Hydrolysates Derived from Cucumaria frondosa Tentacles

#### 2.4.1. DPPH Radical Scavenging Capacity

**Figure 3:**
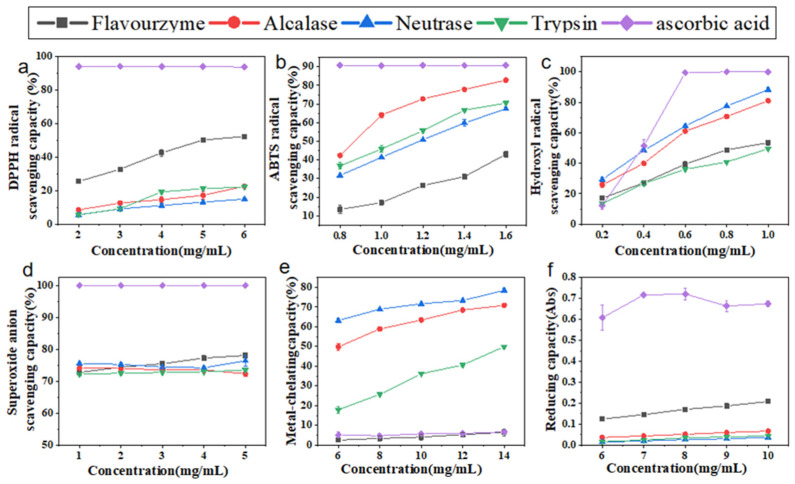
(**a**) DPPH radical scavenging capacity of CFTHs; (**b**) ABTS radical scavenging capacity of CFTHs; (**c**) hydroxyl radical scavenging capacity; (**d**) superoxide anion scavenging capacity of CFTHs; (**e**) metal-chelating capacity of CFTHs; (**f**) reducing capacity of CFTHs (n = 5).

DPPH is widely utilized to assess the antioxidant activity and capability of bioactive substances due to its stability as a free radical [20]. As illustrated in Figure 3a, the scavenging capacity of CFTHs on the DPPH radical exhibited a significant (*p* < 0.05) dose-dependent effect. Among the hydrolysates generated by the four proteases, the hydrolysate derived from flavourzyme demonstrated a significantly higher (*p* < 0.05) DPPH free radical scavenging capacity compared to the other three proteases. This finding suggests that flavorzyme was particularly effective in generating peptides with potent capabilities to scavenge DPPH free radicals, exhibiting an IC_50_ of 5.34 mg/mL, which is consistent with that in the study by Yu et al. [21].

#### 2.4.2. ABTS Radical Scavenging Capacity

The ABTS method is a highly sensitive technique employed to confirm the free radical activity of samples [22]. Consistent with the findings of Hernández-Ruiz et al. [20], the scavenging capacity of CFTHs increased with concentration (Figure 3b). In comparison, the scavenging capacity of alcalase hydrolysate was significantly stronger than that of the other three proteases, with an IC_50_ of 0.82 mg/mL, which aligned with that in the study conducted by Karami et al. [9]. Additionally, the alcalase hydrolysate exhibited a higher content of aromatic amino acids, which can convert free radicals into stable molecules by acting as electron donors. This mechanism enhances the ABTS radical scavenging capacity [23].

#### 2.4.3. Hydroxyl Radical Scavenging Capacity

The hydroxyl radical, known for its high reactivity with biological tissue proteins, can induce cellular damage and contribute to various physiological disorders when it interacts with endogenous biomolecules such as proteins [24]. This study demonstrated that the hydroxyl radical scavenging capacity of CFTHs increased with concentration, ranging from 0.2 to 1.0 mg/mL (Figure 3c). Notably, at lower concentrations, CFTHs exhibited higher scavenging activity compared to ascorbic acid, with the hydrolysate from neutrase displaying the strongest scavenging capacity, characterized by an IC_50_ of 0.44 mg/mL. Furthermore, CFTHs demonstrated a greater hydroxyl radical scavenging capacity than both mung bean protein hydrolysate [23] and hydrolysates of milk protein concentrate [24]. Therefore, CFTHs show promise as a potent scavenger in the food industry.

#### 2.4.4. Superoxide Anion Scavenging Capacity

The superoxide anion scavenging capacity of CFTHs was significantly lower (*p* < 0.05) compared to that of ascorbic acid. Additionally, no significant differences (*p* > 0.05) were observed in the scavenging activity among the four proteases (Figure 3d). The presence of inactive polypeptides in CFTHs may contribute to the reduced clearance activity [25].

#### 2.4.5. Metal-Chelating Capacity

Metal ions play a significant role in promoting lipid peroxidation. Therefore, the removal of these ions from food systems can effectively mitigate oxidation reactions [26]. Hydrolysates produced by alcalase, neutrase, and trypsin exhibited higher chelating activity compared to ascorbic acid, whereas flavourzyme demonstrated a chelating activity comparable to that of ascorbic acid. Alcalase, an endopeptidase with broad specificity, efficiently cleaves polypeptide bonds, thereby increasing the abundance of acidic and basic amino acids, especially phenylalanine (Phe), tyrosine (Tyr), and lysine (Lys). This enhancement in negative charge density accounts for the enhanced chelation activity of polypeptides following enzymatic hydrolysis [27].

#### 2.4.6. Reducing Capacity

The reducing capacity of protein hydrolysates is commonly utilized as an indicator of their potential antioxidant and electron-donating abilities [28]. A higher absorbance at 700 nm indicates a stronger reducing capacity. In comparison to ascorbic acid (Figure 3f), CFTHs exhibited a higher reducing capacity, although it was lower than that of yellowfin tuna skin (*Thunnus albacares*) hydrolysate [29]. The pronounced reducing capacity of flavourzyme may be attributed to the release and exposure of amino acids such as Phe, tryptophan (Try), aspartic acid (Asp), and glutamic acid (Glu). The exposure of these amino acid side chain groups during hydrolysis can provide an additional supply of electrons and protons, thereby maintaining the reducing capacity [30].

### 2.5. Antioxidant Activity Stability of Hydrolysates Derived from Cucumaria frondosa Tentacles

**Figure 4:**
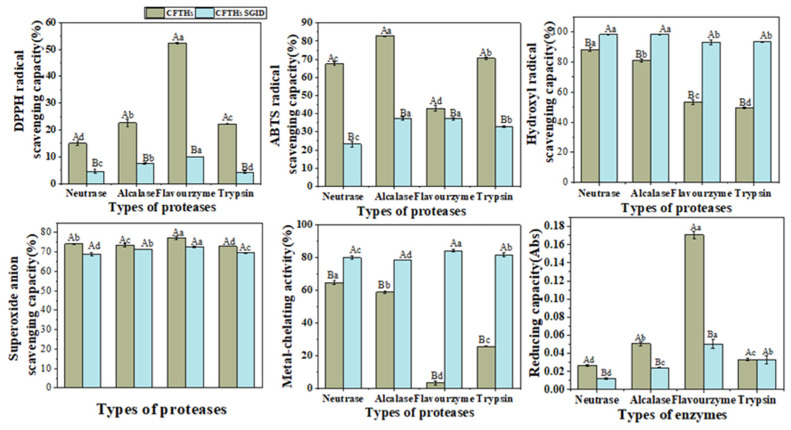
Antioxidant activity stability of CFTHs after in vitro simulated gastrointestinal digestion (n = 5). The uppercase letters indicate significant differences in the activity of CFTHs obtained by the same protease hydrolysis before and after in vitro simulated gastrointestinal digestion, and the lowercase letters indicate significant differences in the activity of CFTHs obtained by different protease hydrolysis.

Considering the potential use of CFTHs as a functional food ingredient, it is crucial to account for appropriate food processing and storage conditions when incorporating them into functional foods. This study examined the antioxidant activity of CFTHs during in vitro simulated digestion. As depicted in Figure 4, the DPPH radical scavenging capacity, ABTS radical scavenging capacity, and reducing capacity of CFTHs decreased following in vitro simulated gastrointestinal digestion. These results align with the findings derived by Lee et al. [31]. However, the hydroxyl radical scavenging capacity and metal-chelating capacity of CFTHs significantly increased, while the superoxide anion scavenging capacity remained unchanged, after in vitro simulated gastrointestinal digestion. This phenomenon can be attributed to the breakdown of larger proteins into hydrolysates, which may result in a persistence of, reduction in or enhancement of antioxidant properties due to the production of smaller peptides and the release of free amino acids [32]. The enhanced antioxidant properties observed after gastrointestinal digestion can be ascribed to the release of peptides and amino acids through the action of pepsin and pancreatic enzymes. A study conducted by Morais et al. [33] reported that small peptides exhibited resistance to hydrolysis by digestive enzymes, and were readily absorbed in the gut. This finding may elucidate why flavourzyme hydrolysates demonstrate greater resistance to gastrointestinal digestion compared to the other three proteases.

**Figure 5:**
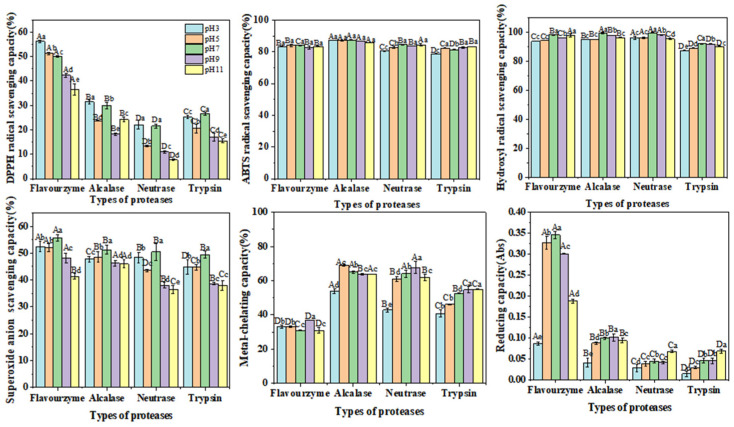
Effects of varying pH levels on the antioxidant activity of CFTHs (n = 5). The uppercase letters indicate significant differences in the antioxidant activity of CFTHs at the same pH level, and the lowercase letters indicate significant differences in the antioxidant activity of CFTHs at different pH levels.

The effect of pH on the antioxidant activity of CFTHs is illustrated in Figure 5. pH is a crucial factor influencing the stability of active peptides. Generally, the DPPH radical scavenging capacity, superoxide anion scavenging capacity, metal-chelating capacity, and reducing capacity were optimal in a neutral environment, which is consistent with the findings of Sheng et al. [34]. In contrast, the ABTS radical scavenging capacity and hydroxyl radical scavenging capacity were not significantly affected by variations in pH. Nevertheless, changes in pH can influence the biological activity of the hydrolysate, potentially related to the presence of specific amino acids. For instance, acidic conditions may lead to the degradation of glutamine and asparagine residues, while alkaline conditions can result in the degradation of threonine, serine, and cysteine residues, thereby altering their biological activity [35].

**Figure 6:**
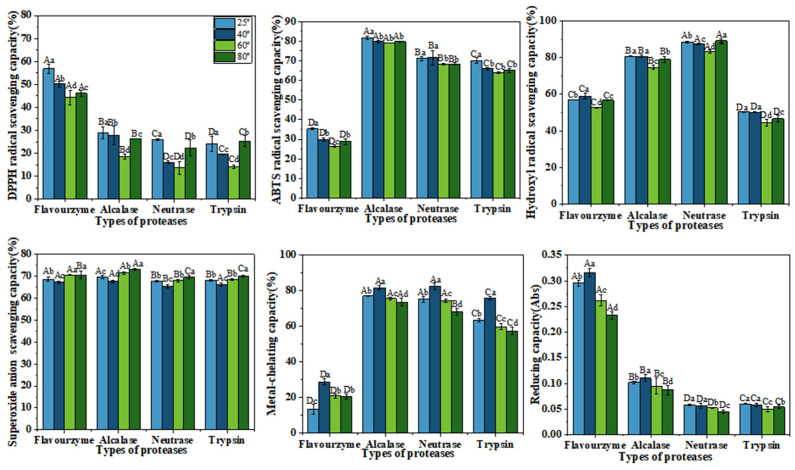
Effects of different temperatures on the antioxidant activity of CFTHs (n = 5). The uppercase letters indicate significant differences in antioxidant activity of CFTHs at the same temperature, and the lowercase letters indicate significant differences in antioxidant activity of CFTHs at different temperatures.

As illustrated in Figure 6, the antioxidant activity of CFTHs displayed slight fluctuations across varying temperatures but remained relatively stable, indicating good thermal stability. These findings are consistent with those reported by Zhang et al. [12]. It is plausible that the low molecular mass of the samples utilized in this experiment contributes to their reduced sensitivity to temperature, resulting in the minimal structural denaturation of the peptides when exposed to temperatures below a certain threshold. Consequently, the observed changes in activity were less pronounced [36].

### 2.6. Effects of Hydrolysates Derived from Cucumaria frondosa Tentacles on Body Weight and Visceral Index in Mice

**Figure 7:**
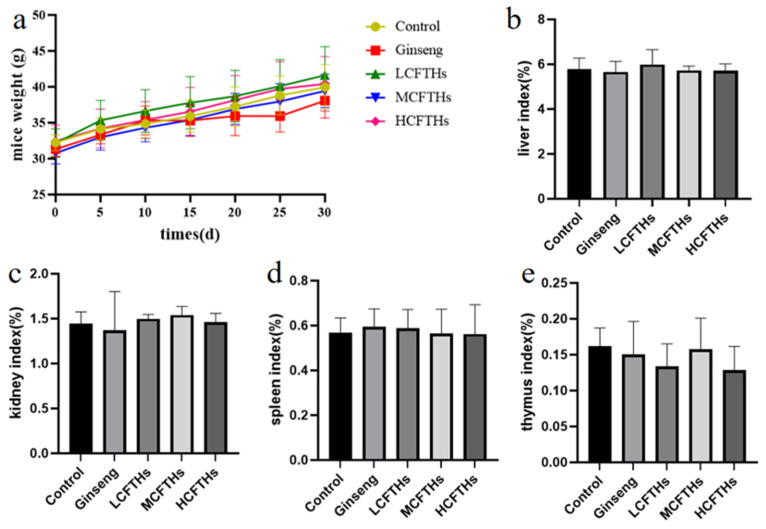
Effects of CFTHs on body weight and visceral index in mice (n = 9). (**a**) Changes in body weight of mice; (**b**) liver index; (**c**) kidney index; (**d**) spleen index; (**e**) thymus index.

Changes in the body weight of mice during gavage can reflect the effects of CFTHs on appetite and overall health status [37]. The body weights of mice after 30 days of continuous gavage are presented in Figure 7. As the duration of intragastric administration increased, the body weight of the mice gradually increased. However, there was no significant difference in the growth rate of body weight among the various groups of mice during the administration period, indicating that CFTHs did not have a notable effect on appetite or health status. Furthermore, there were no significant differences in the coefficients of the liver, kidney, spleen, and thymus between the experimental group and the blank control group (*p* > 0.05). This suggests that the administration of CFTHs at different doses did not impact the visceral index of the mice.

### 2.7. Effects of Hydrolysates Derived from Cucumaria frondosa Tentacles on Mice Liver Tissue Morphology

**Figure 8:**
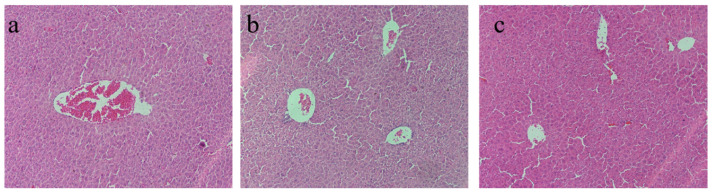
Liver histomorphology of mice. (**a**) Control group; (**b**) positive control group; (**c**) CFTHs group.

Following exhaustive swimming, the liver tissue structure of the mice exhibited varying degrees of damage. In the control group, cell boundaries were indistinct, the cytoplasm appeared fused and swollen, and the nuclei exhibited signs of shrinkage (Figure 8). In contrast, supplementation with CFTHs demonstrated a protective effect on the livers of mice subjected to exhaustive swimming.

### 2.8. Effects of Hydrolysates Derived from Cucumaria frondosa Tentacles on Weight-Loaded Swimming Capacity

**Figure 9:**
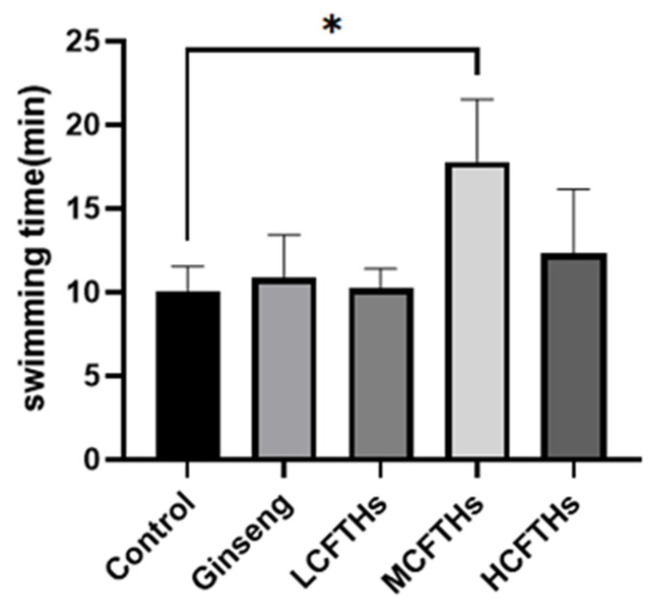
Swimming time of mice (n = 9). * *p* < 0.05.

The forced swimming test is a widely utilized method for assessing the anti-fatigue effects of biologically active substances. Systemic changes induced by exercise-related fatigue can be observed in mice, including immune deficiencies and metabolic disorders. This study employed an exercise swimming test to evaluate the physical fatigue experienced by mice and to assess the anti-fatigue effects of CFTHs. Our results indicate that MCFTHs significantly prolonged the forced swimming time (*p* < 0.05) compared to the control group, with a mean duration of 17.81 min (Figure 9). These findings demonstrate that the MCFTHs-treated groups exhibited enhanced swimming endurance.

### 2.9. Effects of Hydrolysates Derived from Cucumaria frondosa Tentacles on Liver Glycogen and Blood Glucose in Mice

**Figure 10:**
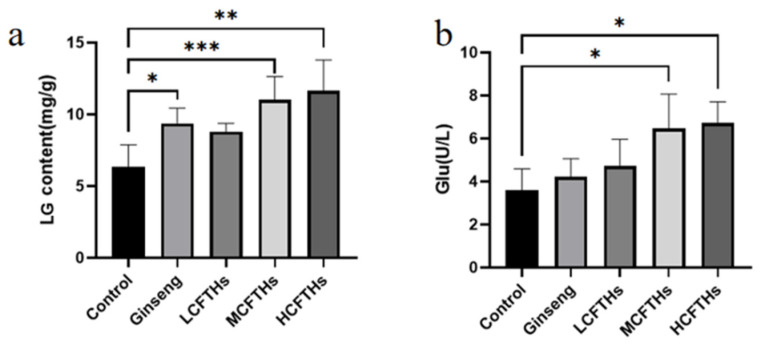
Effects of CFTHs on LG and Glu in mice (n = 9). (**a**) LG content; (**b**) Glu content. * *p* < 0.05, ** *p* < 0.01, *** *p* < 0.001.

Glycogen content serves as a crucial indicator of physical fatigue. As illustrated in Figure 10a, the serum liver glycogen (LG) content in mice was measured, revealing that CFTHs elevated LG levels in a dose-dependent manner compared to the control group, with HCFTHs demonstrating the most pronounced effect, increasing LG by 83.49%. These results indicate that CFTHs could enhance LG reserves, thereby providing energy for the body, alleviating physical fatigue, improving exercise endurance, and exhibiting significant anti-fatigue properties. Glucose serves as the primary energy-supplying substance in the human body, and maintaining stable blood glucose levels is essential for normal physiological functioning and resistance to fatigue. As depicted in Figure 10b, CFTHs significantly increased blood glucose (Glu) levels (*p* < 0.05) compared to the blank control group, with LCFTHs, MCFTHs, and HCFTHs showing increases of 31.82%, 80.52%, and 85.92%, respectively. This indicates that CFTHs could provide glucose to the body, thereby contributing to the alleviation of fatigue.

### 2.10. Effects of Hydrolysates Derived from Cucumaria frondosa Tentacles on Metabolite Accumulation

**Figure 11:**
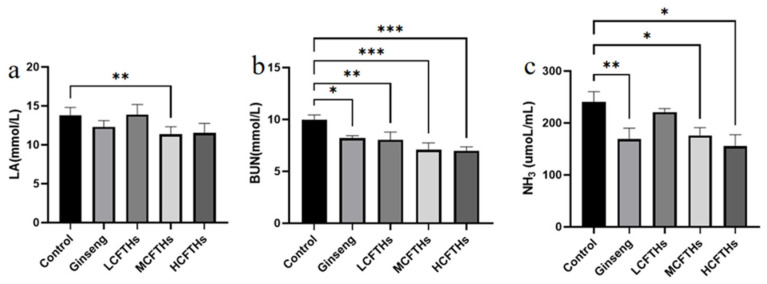
Effects of CFTHs on metabolite accumulation (n = 9). (**a**) LA content; (**b**) BUN content; (**c**) NH_3_ content. * *p* < 0.05, ** *p* < 0.01, *** *p* < 0.001.

The metabolite accumulation theory posits that, as athletes consume greater amounts of energy substrates, the production of metabolites increases, particularly during high-intensity exercise compared to rest. These metabolites accumulate in the blood and skeletal muscle, disrupting the dynamic balance of the internal environment, which subsequently leads to a decline in muscle tissue motor function and results in exercise-induced fatigue [38]. During strenuous exercise, the body experiences the significant depletion of adenosine triphosphate (ATP), accompanied by elevated levels of ammonia (NH_3_) in skeletal muscle. This condition promotes glycolytic reactions, resulting in increased concentrations of lactic acid (LA) and urea nitrogen (BUN). Such disruptions to normal dynamic homeostasis contribute to fatigue. To assess the effects of CFTHs on metabolite accumulation, we measured serum levels of LA, BUN, and NH_3_ in mice. As illustrated in Figure 11, compared to the blank control group, the administration of CFTHs resulted in significant decreases in LA, BUN, and NH_3_ levels, effectively reversing the accumulation of these metabolites. Among the sample groups, the HCFTHs group exhibited the lowest levels of BUN and NH_3_, while the MCFTHs group showed the lowest levels of LA.

### 2.11. Protective Effects of Hydrolysates Derived from Cucumaria frondosa Tentacles on Muscle

**Figure 12:**
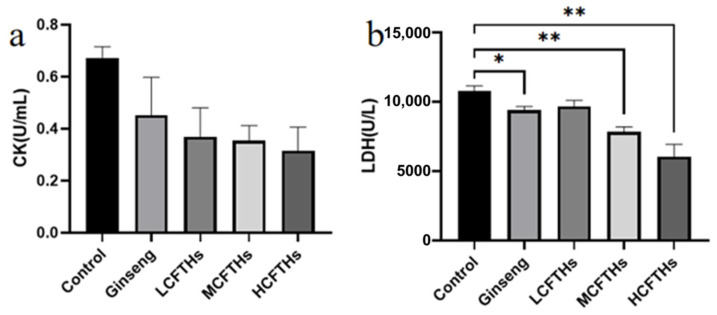
Protective effects of CFTHs on muscle (n = 9). (**a**) CK activity; (**b**) LDH activity. * *p* < 0.05, ** *p* < 0.01.

Creatine kinase (CK) activity is a crucial marker of exercise-induced muscle damage, as CK is prevalent in various muscle cells types, including skeletal, smooth, and cardiac muscles. An increase in CK activity in the bloodstream typically indicates damage to muscle cells, resulting in the release of CK into the circulation [39]. Additionally, lactate dehydrogenase (LDH) serves as an indicator of muscle fiber damage and is primarily located in muscle tissue. Elevated LDH levels often occur following prolonged exercise, leading to its leakage into the bloodstream [40]. As illustrated in Figure 12, CK activity was reduced in the CFTHs group compared to the control group. Furthermore, CFTHs markedly (*p* < 0.01) inhibited the increase in blood LDH concentration induced by forced swimming, suggesting that CFTHs effectively mitigate muscle fiber rupture. These findings suggest that CFTHs can substantially lower CK and LDH levels in the blood, thereby aiding recovery from physical fatigue and providing muscle protection.

### 2.12. Effects of Hydrolysates Derived from Cucumaria frondosa Tentacles on Antioxidant Levels in Mice Liver

**Figure 13:**
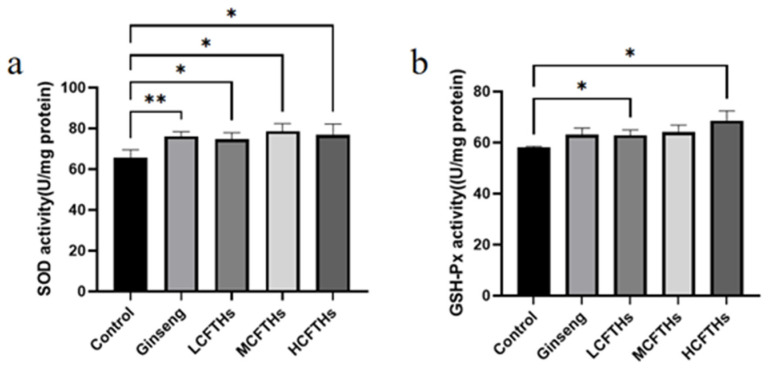
Effects of CFTHs on antioxidant levels in mice liver (n = 9). (**a**) SOD activity; (**b**) GSH-Px activity. * *p* < 0.05, ** *p* < 0.01.

Oxidative stress induced by strenuous exercise may contribute to exercise-related fatigue [41]. Superoxide dismutase (SOD) plays a critical role among antioxidant enzymes in combating oxidative stress. Glutathione peroxidase (GSH-Px) catalyzes the formation of glutathione, which is the primary non-protein thiol in organisms, and is essential for enhancing antioxidant defenses and reacting with ROS to mitigate oxidative damage and lipid peroxidation. Additionally, GSH-Px facilitates the decomposition of hydrogen peroxide into water and oxygen, thereby preventing the deleterious effects of excess hydrogen peroxide on tissues and organs [42]. As illustrated in Figure 13, CFTHs significantly increased the activities of SOD and GSH-Px in mice. Compared to the control group, HCFTHs enhanced the activities of SOD and GSH-Px by 16.91% and 18.29%, respectively. These findings suggest that CFTHs could elevate antioxidant enzyme activities, thereby protecting the body from oxidative damage associated with strenuous exercise.

## 3. Materials and Methods

### 3.1. Materials and Reagents

Recently harvested *Cucumaria frondosa* tentacles were acquired from the Haizhongbao seafood market in Yantai, China. Food-grade neutrase (5.0 × 10^4^ U/g), alcalase (2.0 × 10^5^ U/g), flavourzyme (1.5 × 10^4^ U/g), and trypsin (2.5 × 10^5^ U/g) were sourced from Solarbio biotechnology Co. Ltd. in Beijing, China. All other reagents used in this study were of analytical grade.

### 3.2. Preparation of Hydrolysates Derived from Cucumaria frondosa Tentacles

As shown in Table 1, the tentacles of *Cucumaria frondosa* were subjected to hydrolysis through the addition of proteases to the prepared CFTHs. This hydrolysis process was conducted in a water bath shaker set to a speed of 100 rpm. Following the incubation period, the mixture was heated in a water bath at a temperature of 90 ± 2 °C for 15 min to terminate the hydrolysis process. The resulting CFTHs were then rapidly chilled in ice water and subsequently centrifuged at 10,000 rpm for 15 min at a temperature of 4 °C. The supernatant obtained from centrifugation was freeze-dried to yield the CFTHs.

### 3.3. Determination of the Degree of Hydrolysis

The DH value of CFTHs was determined using a slightly modified ninhydrin colorimetric method [43]. In this procedure, 2 mL of the sample was mixed with 1 mL of the ninhydrin solution in a test tube. The tubes were subsequently heated in a boiling water bath for 15 min. Following rapid cooling in cold water, 5 mL of a 40% ethanol solution was added to the tube. The solution was then vigorously shaken until the brownish-red color faded, after which it was allowed to stand at room temperature for 10 min. The absorbance of the solution was measured at 570 nm, with the baseline established using distilled water. The DH was calculated using the following equation:

(1) $$DH\left(\%\right)=\frac{h}{h_{tot}}\times 100$$

where *h* represents the millimoles of peptide bonds cleaved per gram of sample (mmol) and *h*_tot_ represents the millimoles of peptide bonds present per gram of sample (mmol). The *h*_tot_ value used in this experiment was 8.56 mmol/g, determined based on the amino acid composition of *Cucumaria frondose* tentacles.

### 3.4. Determination of the Solubility

The solubility of CFTHs was determined using the refined method described by Vásquez et al. [44]. Solutions of CFTHs (20 mL at a concentration of 1%, *w*/*v*) were prepared, and their pH values were adjusted to 2.0, 4.0, 6.0, 8.0, and 10.0 using 1 mol/L NaOH or 1 mol/L HCl. Following incubation, the hydrolysate solution was centrifuged at 4000 rpm for 15 min, and the resulting supernatant was collected. The protein content of the supernatant was assessed using the method established by Lowry et al. [45], and the solubility of the samples was calculated as a percentage according to the following equation:

Solubility (%) = (Protein content of the supernatant)/(Protein content of the sample) × 100(2)

### 3.5. Determination of the Particle Size, Polydispersity Index, and Zeta Potential

The particle size, PDI, and zeta potential of CFTHs were determined using a modified version of the method described by Wang et al. [17]. Prior to measurement, each sample was dispersed in 0.01 M sodium dihydrogen phosphate buffer (pH 7.0) at a concentration of 0.3 mg/mL. The dispersion was subsequently analyzed using a laser particle size analyzer (Malvern Mastersizer, Malvern Instruments, Malvern, UK).

### 3.6. Antioxidant Activity

#### 3.6.1. DPPH Radical Scavenging Capacity

The DPPH radical scavenging capacity of CFTHs was measured following the method outlined by Du et al. [46]. Various concentrations of samples (2.0, 3.0, 4.0, 5.0, and 6.0 mg/mL) were combined with DPPH (0.2 mM) in equal proportions and incubated at 25 °C for 30 min in the dark. Following the incubation period, the absorbance was measured at 517 nm, with vitamin C (VC) serving as a positive control. Anhydrous ethanol of the same volume was utilized as a blank control. The DPPH radical scavenging capacity was calculated using the following formula

DPPH radical scavenging capacity (%) = [1 − (*A*_1_ − *A*_2_)/*A*_0_] × 100 (3)

where *A*_0_ represents the absorbance values of anhydrous ethanol and DPPH, *A*_1_ represents the absorbance values of the sample and DPPH, and *A*_2_ represents the absorbance values of anhydrous ethanol and the sample.

#### 3.6.2. Hydroxyl Radical Scavenging Capacity

The hydroxyl radical scavenging capacity was assessed using the method described by Zhou et al. [47], with minor modifications. Samples at concentrations of 0.2, 0.4, 0.6, 0.8, and 1.0 mg/mL were mixed with FeSO_4_ (2 mM) and a salicylic acid–ethanol solution (6 mM) in a 1:1:1 ratio. Subsequently, 1.0 mL of H_2_O_2_ (6 mM) was added to the mixture, which was then incubated in a water bath at 37 °C for 30 min. The absorbance of the mixture was measured immediately at 510 nm using a microplate reader, with VC serving as a positive control. The hydroxyl radical scavenging activity was calculated using the following equation:

Hydroxyl radical scavenging capacity (%) = [1 − (*A*_2_ − *A*_1_)/*A*_0_)] × 100 (4)

where *A*_0_ represents the absorbance value of the blank group, *A*_1_ represents the absorbance value of the sample after replacing H_2_O_2_ with water, and *A*_2_ represents the absorbance value of the sample group.

#### 3.6.3. Superoxide Anion Scavenging Capacity

The scavenging capacity of the superoxide anion was evaluated using a modified version of the method described by Xie et al. [48]. CFTHs were prepared in solutions with concentrations of 1.0, 2.0, 3.0, 4.0, and 5.0 mg/mL. A volume of 0.5 mL from each concentration of the samples was transferred into a test tube, followed by the addition of 5 mL of Tris-HCl buffer (50 mM, pH 8.2). The mixture was incubated at 25 °C for 20 min. For the blank group, an equivalent volume of distilled water was added. In the determination tube, the VC standard was substituted with the sample solution. Subsequently, 0.3 mL of a 10 mM pyrogallol solution was introduced. After a reaction period of 3 min, the absorbance was measured at 325 nm. The scavenging capacity of the superoxide anion (O^2−^) was calculated using the following formula:

Superoxide anion radical scavenging capacity (%) = (1 − *A*_1_/*A*_0_) × 100 (5)

where *A*_1_ represents the absorbance value of the sample group and *A*_0_ represents the absorbance value of the blank group.

#### 3.6.4. Reducing Capacity

The reducing capacity was determined according to the method described by Liu et al. [49], with several modifications. Samples of varying concentrations (1.0 mL, 6.0–10.0 mg/mL) were incubated with 1.0 mL of 1% K_3_Fe(CN)_6_ and 1.0 mL of 0.2 M PBS buffer (pH 6.8). Following a reaction at 50 °C for 20 min, 1.0 mL of 10% TCA solution was added to the mixture. The resulting solution was then centrifuged at 5000 rpm for 10 min, and 2.5 mL of the supernatant was extracted. Following this, 2.5 mL of deionized water and 1.2 mL of 0.1% FeCl_3_ solution were added sequentially. The absorbance of the resulting complex was measured at 700 nm, with higher absorbance values indicating a stronger reducing capacity.

#### 3.6.5. Metal-Chelating Capacity

The metal-chelating capacity was evaluated using the method outlined by Gao et al. [50], with some modifications. A 0.50 mL sample of CFTHs, with concentrations ranging from 6.0 to 14.0 mg/mL, was mixed with 2 mL of water, 5 μL of FeCl_2_ (2 mM), and 100 μL of ferrozine (5 mM). The mixture was shaken and allowed to stand at 25 °C for 10 min. Subsequently, the absorbance was measured at 562 nm. The absorbance of the blank was determined using the same procedure, substituting distilled water for the sample. The metal-chelating ability was calculated as follows:

Metal-chelating capacity (%) = [1 − *A*_1_/*A*_0_] ×100 (6)

where *A*_1_ represents the absorbance value of the sample group, and *A*_0_ represents the absorbance value of the blank group.

#### 3.6.6. ABTS Radical Scavenging Capacity

According to Liu et al. [49], CFTHs at various concentrations (0.2, 0.4, 0.6, 0.8, and 1 mg/mL) were mixed with the ABTS diluent in a ratio of 1:8 and incubated at 37 °C for 10 min. The absorbance was measured at 734 nm using a microplate reader (Thermo Scientific, Waltham, MA, USA). The ABTS radical scavenging capacity was calculated using the following formula:

ABTS radical scavenging capacity (%) = (1 − *A*_1_/*A*_0_) × 100 (7)

where *A*_0_ represents the absorbance value of the blank group, and *A*_1_ represents the absorbance value after the addition of the sample.

### 3.7. Determination of Antioxidant Activity Stability

#### 3.7.1. Simulated Digestion In Vitro

According to Minekus et al. [51], the method of in vitro simulated digestion of hydrolysates was employed. All hydrolysate solutions were maintained at an equivalent protein concentration.

Gastric digestion stage: The sample was dissolved in water (520 mg/5 mL) and combined with 7.5 mL of simulated gastric juice, 1.6 mL of pepsin (resulting in a final enzyme concentration of 2000 U/mL, as configured by the simulated gastric juice), 5 μL of 0.3 M CaCl_2_, and 695 μL of water. The pH of the mixture was adjusted to 3.0 ± 0.1 using 1 M HCl. The beaker was then placed on a magnetic stirrer at 37 °C and 200 rpm for 2 h. Upon completion of the simulated digestion, the pH was adjusted to 7 to terminate the action of the protease.

Simulated intestinal digestion stage: A 20 mL aliquot of the gastric phase digestion mixture was combined with 11 mL of simulated intestinal fluid, 5 mL of trypsin (resulting in a final enzyme concentration of 100 U/mL, as configured by the simulated intestinal fluid), 2.5 mL of bile salt (10 mM), 40 μL of 0.3 M CaCl_2_, and 1.31 mL of water. The pH of this mixture was adjusted to 7.0 ± 0.1 using either 1 M NaOH or 1 M HCl. The beaker was again placed on a magnetic stirrer at 37 °C and 200 rpm for 2 h. Following the reaction, the mixture was precooled in ice water for 10 min and subsequently frozen at −40 °C for an additional 10 min to halt the trypsin reaction.

#### 3.7.2. Temperature and pH Stability Test

The stability of the antioxidant activity of the hydrolysates was evaluated using a modified method based on the work of Xiang et al. [52]. The hydrolysate solutions, maintained at an equivalent protein concentration, were incubated at temperatures of 25, 40, 60, and 80 °C for a duration of 2 h. Following incubation, the samples were cooled to room temperature, and their antioxidant activity was evaluated. To investigate the impact of pH on the stability of antioxidant activity, the pH of the hydrolysate was initially adjusted to a range of 3.0 to 11.0 and allowed to incubate at room temperature for 2 h. Following this incubation period, the pH was readjusted to 7.0, and the antioxidant activity was subsequently assessed.

### 3.8. Animal Experiments

Ninety male ICR mice, each weighing 26 ± 2 g, were purchased from Jinan Pengyue experimental animal breeding Co., Ltd. (Jinan, China). The environmental conditions were maintained at a room temperature of 25 ± 2 °C, with a relative humidity of 50 ± 5%, and a 12 h light/12 h dark cycle. The mice were routinely fed a standard diet and had free access to food and water. During the week of adaptive feeding, the mice underwent training sessions twice to acclimatize them to swimming for 5 min each on days 2 and 5, respectively. Following a one-week adaptation period, all mice successfully learned to swim and were then randomly divided into five groups, with 18 mice in each group, distributed across four or five cages. The five experimental groups included a blank control group (receiving distilled water of the same volume), a positive control group (receiving ginseng at a dosage of 0.5 mg/g body weight/day), a low-dose CFTHs group (LCFTHs, 0.25 mg/g body weight/day), a medium-dose CFTHs group (MCFTHs, 0.5 mg/g body weight/day), and a high-dose CFTHs group (HCFTHs, 1 mg/g body weight/day). Each mouse was administered the corresponding dose of the test substance daily, based on its body weight, using a gavage volume of 0.01 mL/g. This was conducted once a day for 30 consecutive days with the aid of oral gavage needles. The body weight of the mice was measured daily throughout the trial. At the conclusion of the study, cervical dislocation was performed for euthanasia following blood collection from all mice. Additionally, the weights of the liver, kidney, spleen, and thymus were recorded, and the visceral index was calculated by dividing the visceral weight (in grams) by the body weight of each mouse (in grams).

### 3.9. The Exhaustive Swimming Experiment

Thirty minutes after the last administration, nine mice were randomly selected from each group, and their body surface grease was removed using soapy water. A 5% body weight lead weight was affixed to the base of each mouse′s tail, after which the mice were placed in a constant temperature water bath. The swimming test was conducted in a bathtub maintained at 25 °C with a length of 100 cm, 75 cm in width, and filled with water to a depth of 60 cm. Timing commenced as the mice began to swim and concluded when a mouse exhibited exhaustion, defined as a noticeable loss of coordinated movements and an inability to return to the water surface within at least 10 s. At this point, timing was stopped to record the exhaustive swimming time. During the swimming period, if any mouse ceased or suspended its movement, a glass rod was employed to stir the surrounding water to encourage continuous movement. At the conclusion of the study, cervical dislocation was performed for euthanasia.

### 3.10. Hematoxylin–Eosin Staining

The liver sample from the mouse was excised and immediately placed in 4% formalin for 24 h. Following fixation, the sample underwent dehydration through a series of gradient ethanol solutions, ranging from 70% to 100%. It was then embedded in paraffin using a tissue embedding technique. Subsequently, the tissue was sectioned into 4 μm slices using a microtome and stained with hematoxylin and eosin. Photographs were captured using an optical microscope equipped with a camera (Nikon Eclipse CI, Tokyo, Japan).

### 3.11. Determination of Serum and Liver Biochemical Parameters

Thirty minutes after the final administration, the mice were placed in a swimming box with a water temperature maintained at 25 ± 1 °C and a minimum water depth of 30 cm. Following 30 min of swimming, blood samples were collected and centrifuged to obtain serum, which was subsequently analyzed for Glu content, LDH, CK, LA, BUN, and NH_3_.

Following serum collection, the mice were immediately sacrificed. A suitable amount of mouse liver was combined with an appropriate volume of normal saline to prepare a 10% liver tissue homogenate. This homogenate was ground and then centrifuged at 4000 rpm for 20 min to obtain the supernatant. A kit was utilized to assess the levels of LG in the mouse liver, as well as the activities of SOD and GSH-Px in the liver tissue.

### 3.12. Statistical Analysis

All experimental data are presented as mean ± standard deviation. One-way ANOVA, followed by Tukey’s test (for equal variances) or Dunnett’s T3 test (for different variances), was employed when the samples were normally distributed. In contrast, the Kruskal–Wallis test was utilized for samples that were not normally distributed. Statistical analyses were conducted using GraphPad Prism 9.0, and a *p*-value of less than 0.05 was deemed statistically significant. Graphs were generated using Origin 2018 (Origin Lab Corporation, Northampton, MA, USA).

## 4. Conclusions

This study investigated the characteristics, antioxidant activity stability, and anti-fatigue effects of hydrolysates derived from the tentacles of *Cucumaria frondosa* using four different proteases—flavourzyme, alcalase, neutrase, and trypsin. The results indicate that the hydrolysates produced by flavourzyme exhibited a higher DH compared to those generated by the other proteases. Additionally, the alcalase hydrolysate displayed decreased dispersibility in aqueous solution. The hydrolysates obtained through protease hydrolysis demonstrated significant antioxidant capacity, with flavourzyme hydrolysates showing enhanced stability during gastrointestinal digestion. Furthermore, the CFTHs exhibited no toxic effects on mice, with a consistent increase in body weight observed during intragastric administration. The anti-fatigue effect of the CFTHs was significant, as evidenced by an extension in the exhaustive swimming time of the mice. The CFTHs also enhanced the levels of LG and Glu. Moreover, the CFTHs provided a protective effect on muscle tissue and reduced the accumulation of metabolites such as LA, BUN, NH_3_, CK, and LDH. The levels of SOD and GSH-Px in the CFTHs groups were elevated, indicating that the CFTHs inhibited exercise-induced fatigue. These findings offer substantial reference value for the development of CFTHs as functional foods with significant anti-fatigue properties. Additionally, they present potential opportunities for the high-value utilization of *Cucumaria frondosa* tentacles, which could yield considerable ecological and economic benefits.

## Figures and Tables

**Table 1:** Optimal enzymatic hydrolysis conditions of different proteases.

Proteases	Temperature (°C)	pH	Solid–Liquid Ratio (*w*/*v*)	Proteases Addition (U/g Protein)	Time (h)
Flavourzyme	40	5.8	1:6	10,000	7
Alcalase	55	9.5	1:8	8500	7
Trypsin	55	8	1:4	9500	4
Neutrase	45	7.2	1:10	7500	5

## Data Availability

The datasets generated and analyzed during the current study are available from the corresponding authors upon reasonable request.
